# Examining the Influence of Exploration and Parental Education Attainment on Students’ Acceptance of Collectivist Values

**DOI:** 10.3390/ejihpe13070094

**Published:** 2023-07-13

**Authors:** Ruining Jin, Tam-Tri Le, Minh-Hoang Nguyen, Quan-Hoang Vuong

**Affiliations:** 1 Civil, Commercial and Economic Law School, China University of Political Science and Law, Beijing 100088, China; 2 Centre for Interdisciplinary Social Research, Phenikaa University, Hanoi 100803, Vietnam; hoang.nguyenminh@phenikaa-uni.edu.vn (M.-H.N.); hoang.vuongquan@phenikaa-uni.edu.vn (Q.-H.V.); 3 A.I. for Social Data Lab (AISDL), Vuong & Associates, Hanoi 100000, Vietnam

**Keywords:** collectivist values, exploration, parental education, information processing, Bayesian Mindsponge Framework

## Abstract

Exploration can help students access a wider range of information and make connections among values within the natural and social world. This study investigated the relationship between students’ previous exploration of their surroundings and their acceptance of collectivist values in the context of China. A sample of 343 college students was analyzed based on the Bayesian Mindsponge Framework to explore this relationship. The results revealed a positive association between students’ prior exploration of surroundings and their degree of collectivist orientation. Furthermore, parental education attainment was found to negatively moderate this association, albeit with a small effect size. These findings contribute to the understanding of how information acquisition influences students’ acceptance of collectivist values and highlight the potential role of the family infosphere in shaping this relationship.

## 1. Introduction

Collectivism is a sociocultural term that refers to the prioritization of group goals, values, and interests over those of individual members [1]. Collectivist cultures emphasize the interdependence of individuals within a group, such as families, communities, or organizations, and often place a high value on conformity, cooperation, and harmony among group members [2]. Despite these positive properties, collectivist cultures may have certain undesirable effects, such as suppressing innovation, lack of individual rights, and in-group favoritism [1,3,4]. Members in a collectivist society generally tend to spend more time and energy thinking about others, resulting in a culture with a relatively stronger sensitivity to group concerns and consideration for others. Therefore, countries with relatively stronger collectivist values may be considered to be more efficient in fostering collectivist mindsets and implementing policies in a cohesive manner, which helps promote social changes such as progressive taxation and climate change mitigation policies [5].

China is one of the countries that are historically predominated by collectivist cultural values [6]. However, as China went through substantial economic growth since the socioeconomic reform in the 1980s, Chinese society has gradually become more individualized [7]. As trade-offs of economic growth and the surging popularity of individualism, the issues of common benefits, especially social inequality and environmental degradation, began to surface. On the inequality front, the Gini Index of China in 2021 was 0.46—surpassing the United Nations’ warning level of 0.4 [8], and the urban-rural and regional income disparity is also significant [9,10]. On the environmental front, China was ranked 120th out of 180 countries in 2020 on the Environmental Performance Index [11]. In World Air Quality Report 2020, “only 2% of the 388 Chinese cities included in this report achieved the WHO annual PM2.5 target of <10 μg/m^3^” [12]. In the wake of these challenges, while specific measures are needed to handle these issues technically, building positive collectivist values in the mindsets of the younger generation through education is also suggested to be helpful because collectivism can lead to a greater sense of responsibility among individuals, particularly toward their in-group members [1]. Once collectivist values are properly perceived, individuals may be more likely to assist other in-group members over self-interests due to their strong sense of responsibility and commitment to group welfare [1,2,4,13]. In an infosphere containing positive collective norms, progressive policies addressing common benefits such as social inequality and environmental protection would probably face less resistance. Thus, it is important to have a better understanding of the information processes involving one’s perceptions of collectivist values in society.

Exploring one’s surroundings, including both social and physical exploration, can enhance information accessibility to a wider collective infosphere. The Chinese phrase *探索周围环境* (pinyin: *tansuo zhouwei huanjing*) has a textual translation of “exploring the surrounding”. While 探索 (exploring) has a similar connotative use in Chinese, the Chinese use of 周围环境 (the surrounding) has a wider range of implications; it can denote physical (natural or engineered), social, organizational, or even political surrounding environments. From the standpoint of information processing, exploration is one’s interactions with the external infosphere, involving both physical and mental feedback as inputs for internal filtering processes. In the scope of our study, one’s surrounding environments are treated as sources of external information in general, where physical and social explorations are subsets of this broader notion.

Social exploration involves interacting with people from diverse backgrounds, participating in various cultural experiences, and seeking to understand different viewpoints. Such exposure can help individuals recognize the commonalities they share with others, leading to an expanded sense of in-group awareness as individuals begin to connect their subjective values to a broader range of people and appreciate the shared human experiences that transcend cultural and social boundaries [14]. Physical exploration, on the other hand, refers to individuals’ affective responses and physical interactions with the objective world. In this group, natural exploration (exploring natural, non-artificial elements) is a popular concept, which can encompass activities such as hiking, birdwatching, or simply spending time outdoors [15]. In addition to physical interactions, virtual exploration is now also included in natural exploration, thanks to the development of technology. The use of computer-generated imagery (CGI), interactive videos (IR), virtual reality (IR), and augmented reality (AR) have the potential to provide new and innovative ways for students to engage in various virtual natural explorations [16,17,18,19]. It is suggested that exploration can help cultivate perceptions of social connectedness, empathy, and prosocial behaviors [20,21]. Such positive social values, in turn, can support information exchange reinforcement in social connections [22,23,24]. Previous studies have also indicated that individuals with greater natural exploration were more likely to develop pro-environmental attitudes, exhibit concern about living conditions, and engage in pro-environmental behaviors [15,25,26,27].

In addition to exploration, other factors could also contribute to the young generation’s individualism and collectivism, such as parental educational attainment and parenting styles. Generally speaking, the most common parenting style in China is authoritarian parenting (parents’ high demand and low responsiveness) due to its subjectively perceived effectiveness in promoting academic success [28]. In addition to authoritarian parenting, there are also authoritative parenting (marked by high demand and high responsiveness), permissive parenting (characterized by low demand and high responsiveness), and neglectful parenting (low demand and low responsiveness) [29]. However, it is reported that there is a growing trend that the parenting style is shifting from authoritarian to more authoritative and permissive parenting styles in China due to a number of factors, including the increasing availability of information about child development and parenting practices, as well as the growing number of Chinese parents who have themselves been exposed to Western parenting styles [30]. The combination of authoritative and permissive parenting is also called democratic parenting [31,32], which is considered a more progressive parenting style for promoting academic excellence and strong social skills [33,34]. In the context of Chinese middle schools, one study suggested that Chinese parents with higher social status are more likely to adopt a permissive parenting style that respects children’s choice and allow them to act based on how they want [35]. Other studies have also supported this association that the higher socioeconomic status that parents have, the more democratic parenting styles they tend to maintain, and the more individualistic children are likely to be [36,37]. However, there is also a study suggesting that no significant correlation was found between authoritarian parenting and family connectedness [38]. It is possible that the effects of parental education attainment are the results of more complex non-linear information processing pathways.

To further explore the psychological processes of how exploration and parents’ general knowledge levels may influence the ideation of collectivist values, the information processing approach can be useful. In this study, we employed Bayesian Mindsponge Framework (BMF) analytics [39] to achieve this purpose. Detailed rationales for using BMF analytics are presented in the Methodology section. The research objectives in this study are as follows.

- To examine whether Chinese college students’ collectivist value acceptance is associated with their prior exploration experience.
- To examine whether the associations between students’ collectivist value acceptance and prior exploration experience are moderated by parental education attainment.

## 2. Methodology

### 2.1. Theoretical Foundation

#### 2.1.1. Overview of the Mindsponge Theory

The term “mindsponge mechanism” is proposed by Quan–Hoang Vuong and Nancy K. Napier in their studies regarding acculturation and globalization [40]. During the dynamic process of cultural assimilation, the mindset accepts new cultural norms and discards waning ones according to different situations. The information-processing perspective of the mindsponge mechanism can be used to help further elaborate applications following theories in sociology and psychology such as those of Abraham Maslow [41], Geert Hofstede [42], Nonaka and Konno [43], Henry Mintzberg [44], Icek Ajzen [45], and Michael Porter [46], etc.

The mindsponge mechanism has been further developed into a theory of information processing [47]. In this theory, the mind is seen as a collection-cum-processor of information when interacting with the surrounding infosphere. A mindsponge information process has the following characteristics:

(1) It expresses the natural patterns of the biosphere.
(2) It functions through dynamically balanced processes.
(3) It utilizes cost–benefit analysis, which targets the maximization of the perceived benefit of the system and the minimization of its perceived cost.
(4) It uses energy for functioning; thus, energy conservation is a natural prioritization.
(5) It establishes objectives and priorities based on its system’s requirements.
(6) Its essential goal is to extend the existence of the system via survival, growth, and reproduction.

The mindset consists of the accepted information held within the system’s memory. During the information processing procedure, the filtering system based on cost–benefit analysis governs the entry and departure of information, considering the context of the current mindset. The mindset’s trust/distrust of information sources has a major influence on this filtering process. Namely, by utilizing the trust mechanism, one can speed up the decision-making process to allow the entry/departure of information while also preserving energy.

#### 2.1.2. Mindset and Information Filtering

A mindset is a collection of processed information (or trusted values) according to the mindsponge framework. Each mind is distinct because each person’s mindset is unique to a certain degree. Memory, or the capacity to retain information, is the foundation of a mindset. The processing system derives responses regarded as appropriate for the current context from this collection of trusted values. The outputs of conscious and non-conscious mental processes (such as beliefs, thoughts, attitudes, emotions, behaviors, etc.) in humans are determined by the mindset’s existing trusted values. Trusted values are stored in the mindset and serve as references for decision-making when evaluating incoming information. In other words, information that is compatible with the mindset will be allowed to enter the mindset and become new trusted values, while noncompatible information will be rejected. Therefore, during this ongoing interaction between the mind with the surrounding environment, the mindset is updated continuously. The filtering system’s functionality (providing references to evaluate new information) is determined by the mindset. As a result of the changes in mindset, new values are filtered differently, creating a feedback loop for the entire system [47].

All adaptation processing in the human mind is influenced by fundamental physiological properties and the pathways by which humanity has survived as biological organisms and evolved into a social species. The functions of the human brain are therefore governed by the fundamental principles of biochemical processes, which are dependent on the activity of neurons and their synapses. Our social perceptions and behaviors are influenced by distinct information-processing regions of the cerebral cortex [48]. Neuroplasticity enables and regulates dynamic cognition and, as a result, its products, which are thought, emotion, perception, attitude, behavior, etc. [49,50]. Human decision-making and other cognitive functions are determined by the coordination of multiple brain regions, taking into consideration conscious and subliminal mental processes, including reasoning and emotion [51]. The information-filtering mechanisms of the mind reflect the natural evolutionary trends and biosphere component characteristics. The activities and directions of adaptation in any biological system are dependent on consumable resources, particularly usable energy [52]. Consequently, the outcomes of thought processes are constrained by input limitations in the living environment, including physiological aspects (such as physical boundaries, instincts, sensory perceptions, etc.) and social aspects (such as norms, belief systems, education, etc.). The circuitry of the human brain cortex has evolved to optimize energy consumption for conducting complex computational functions (in order to perform well in suboptimal information conditions) [53]. Under limited information input, the mind continuously seeks subjectively optimal solutions from available information sources and references.

A filtering process is summarized below:

(1) The buffer zone is a conceptual space where the mind temporarily stores information obtained from the surrounding environment or memory. The filtering system works to assess the information here.
(2) During the filtering process, the mind uses subjective judgments to determine whether to accept or discard this information based on its perceived costs and benefits. If the perceived benefits outweigh the perceived costs, then the information will be allowed to enter the mindset, and vice versa. Previous trusted values stored in memory are used as references for comparison and connection to the currently evaluated information.
(3) Once accepted, the information will be allowed to enter the mindset and become a new trusted value. Therefore, it can be used as a reference for future assessments of related information and thus will influence the formation of related thoughts, emotions, and behaviors [54].

#### 2.1.3. Model Conceptualization

The mindsponge theory and BMF analytics have been systematically and effectively applied in some former studies in the fields of education [55,56,57] and information absorption from external environments [58,59,60,61,62]. Collectivist values are accepted to exist within the mind if they are perceived to be beneficial based on subjective evaluations. Here, the perceived net value of common properties must be positive, regardless of possible conflicts with self-interests. Due to such perceptions of common benefits, the embracement of collectivist values can be derived from but not limited to group concern sensitivity and environmental awareness. In this regard, previous exploration (both social and natural ones) would result in a better understanding of common properties. Therefore, it is assumed that the more previous explorations students have, the more likely they will accept collectivist values into their mindset, affecting subsequent attitudes and behaviors accordingly. In addition, high parental education attainment has an association with relatively more democratic parenting, which might lead to a higher preference for individualism [37,63]. Frequent interactions with parents lead to parental ideologies influencing the immediate surrounding infosphere. The children likely absorb such ways of thinking, which affects how they interpret the meaning of information acquired from exploration. Hence, from a mindsponge information processing perspective, students with a relatively higher degree of individualistic beliefs would be more likely to filter and reject information containing collectivist values because it is incompatible with the students’ pre-existing individualistic values in the mindset. In this case, information acquired during exploration involving common properties and corresponding collectivist benefits, including the underscoring of sensitivity to group concerns and environmental protection awareness that are associated with collectivist values, could face more resistance during the filtering process because of the incompatibility with individualistic values. As a result, it is assumed that parental education attainment would negatively moderate the possible correlation between students’ previous exploration and collectivist values formation.

### 2.2. Model Construction

#### 2.2.1. Variable Description

The present research utilized a dataset examining the efficacy of motivational interviewing among peers as a strategy to improve career adaptability in at-risk Chinese college students majoring in foreign languages [64]. In order to ensure the appropriate selection and assessment of participants, as well as to evaluate the effectiveness of motivational interviewing, the study utilized the Career Adapt-Abilities Scale—China form [65]. This scale comprises twenty-four items, divided into four dimensions: career concerns (e.g., “Contemplating my future”), career control (e.g., “Making autonomous decisions”), career curiosity (e.g., “Actively seeking personal growth opportunities”), and career confidence (e.g., “Efficiently accomplishing tasks”). Participants rated their responses on a five-point Likert scale, ranging from 1 (“not strong”) to 5 (“very strong”). An invitation for the study was emailed to students studying in the Department of Foreign Languages from six universities in Jilin Province, China. 343 first-year college students (225 women, 118 men; M age = 18.35, SD age = 0.75) volunteered for screening. The research adhered to the principles outlined in the Declaration of Helsinki and received approval from the Ethics Review and Research Committee at the School of Psychology, Northeast Normal University of China, under Protocol #201707. Prior to participating in the study, all individuals provided informed consent. In the case of participants under 18 years of age, consent from their parents or legal guardians was obtained before data collection. The final dataset underwent peer review before being published in an open-access repository. More details of data collection and data description are openly available in the original data article (https://doi.org/10.1016/j.dib.2023.108982 (accessed on 4 March 2023)). The description of variables is presented in Table 1.

The variable *Exploration* represents the magnitude of students’ social and natural exploration of their surroundings prior to the questionnaire. The variable was generated by asking to what degree they agreed with the statement “exploring my surroundings”. The variable was measured on a Likert scale ranging from 1 to 5, with “1” being “not strong”, and “5” indicating “very strong”.

The variable *MotherEdu* represents the mother’s educational attainment. The variable was measured by a scale ranging from 1 to 3, with 1 being junior middle school or below, “2” being a high school diploma or technical secondary education, and “3” being a college degree or above. The variable *FatherEdu* represents the father’s educational attainment of the students, measured in a similar way as *MotherEdu*. The variable *ParentalEdu* is generated, with its value being the sum value of the father’s and mother’s education attainment divided by 2.

The *CollectivistValue* variable reflects the degree of integrating collectivist values. The variable was generated by asking to what degree the students agreed with the statement: “I care about the collective gains and losses as much as my own”. The response is measured on a Likert scale ranging from 1 to 5, with “1” being completely disagree and “5” being completely agree.

#### 2.2.2. Models for Statistical Analysis

Based on the information-processing-based conceptualization above, we formulate Model 1 to examine the possible influence of *Exploration* on *CollectivistValue* and the possible moderating effect of *ParentalEdu* on the aforementioned relationship. Model 1 was constructed as follows:

(1.1) $$ColletivistValue\;\sim \;normal\left(\mu ,\sigma \right)$$

(1.2) $$\mu_{i}=\beta_{0}+\beta_{Exploration}*Exploration_{i}+\beta_{Exploration*ParentalEdu}*Exploration_{i}*ParentalEdu_{i}$$

(1.3) $$\beta \;\sim \;normal\left(M,S\right)$$

Regarding the outcome variable *CollectivisitValue*, the probability around μ is in the form of a normal distribution with standard deviation σ. Participant i’s degree of integrating collectivist values is indicated by $\mu_{i}$. Participant i has an exploration degree of $Exploration_{i}$ and the average parental education attainment value of $ParentalEdu_{i}$. Model 1 has an intercept, $\beta_{0}$, and coefficients, $\beta_{Exploration}$ and $\beta_{Exploration*ParentalEdu}$. Regarding the coefficients, the probability around M is in the form of a normal distribution with standard deviation S.

**Figure 1:**
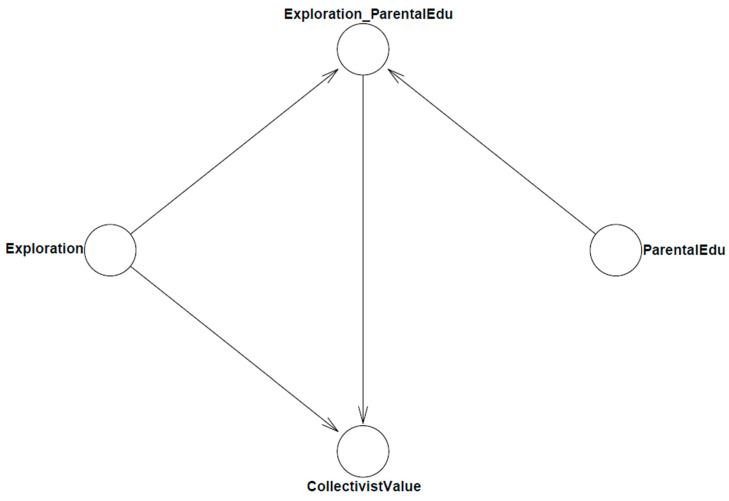
Model 1’s logic network.

The model’s logical network is shown in Figure 1.

In addition to model 1, we want to examine the possible moderating effect of the father’s and mother’s education attainment separately. Thus, we construct Model 2 and Model 3 as follows:

(2.1) $$CollectivistValue\;\sim \;normal\left(\mu ,\sigma \right)$$

(2.2) $$\mu_{i}=\beta_{0}+\beta_{Exploration}*Exploration_{i}+\beta_{Exploration*FatherEdu}*Exploration_{i}*FatherEdu_{i}$$

(2.3) $$\beta \;\sim \;normal\left(M,S\right)$$

(3.1) $$CollectivistValue\;\sim \;normal\left(\mu ,\sigma \right)$$

(3.2) $$\mu_{i}=\beta_{0}+\beta_{Exploration}*Exploration_{i}+\beta_{Exploration*MotherEdu}*Exploration_{i}*MotherEdu_{i}$$

(3.3) $$\beta \;\sim \;normal\left(M,S\right)$$

**Figure A1:**
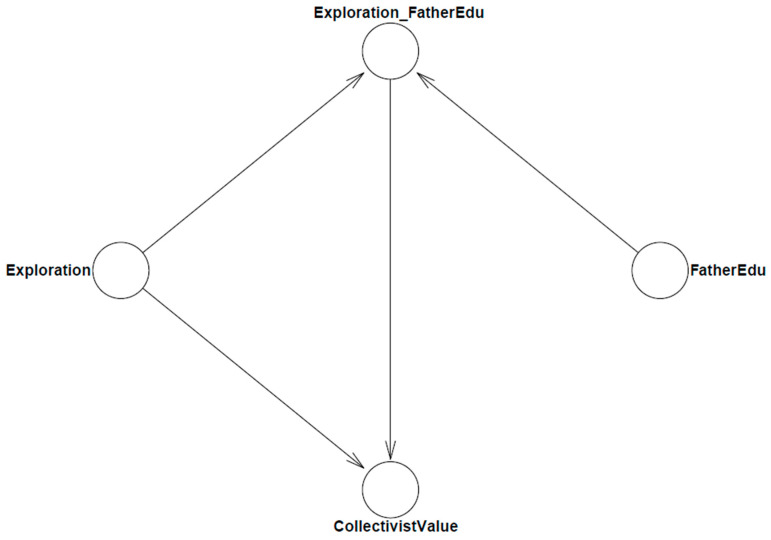
Model 2’s logic network.

**Figure A2:**
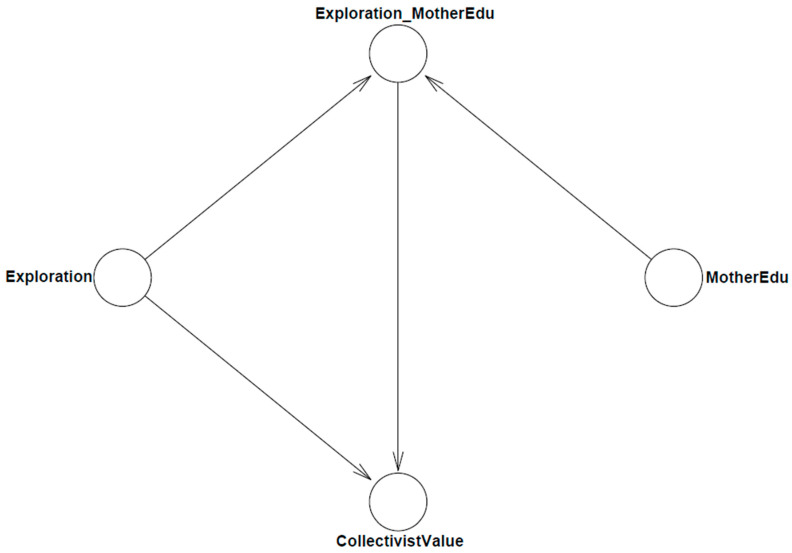
Model 3′s logic network.

Participant i’s father and mother have the education attainment of $FatherEdu_{i}$ and $MotherEdu_{i}$, respectively. The Model 2 and Model 3′s logical networks are shown in Figure A1 and Figure A2 (see Appendix A), respectively.

#### 2.2.3. Statistical Analysis

Following the protocol of BMF analytics, the Bayesian analysis with the Markov chain Monte Carlo (MCMC) technique [39,66] was employed. The aided MCMC algorithms [67,68] help increase the predicting power and accuracy of the Bayesian analysis. The MCMC techniques iteratively generate large samples of the serially correlated parameters drawn from the model’s parameters’ joint posterior distribution. There are some advantages of using the Bayesian approach in this study. Firstly, Bayesian inference sees all properties as probabilistic, including unknown parameters, which is especially advantageous when working with small sample sizes as well as for parsimonious model construction [69,70,71]. Secondly, the Bayesian approach helps avoid the potential risk of over-dependence on the *p*-value, as in the frequentist approach.

Regarding statistical validation, this study evaluates the goodness-of-fit of the models through the Pareto-smoothed importance sampling leave-one-out (PSIS-LOO) diagnostics [72,73], computed as follows:

$$LOO=-2LPPD_{loo}=-2\overset{n}{\underset{i=1}{\sum }}\log\int p\left(y_{i}|\theta \right)p_{post\left(-i\right)}\left(\theta \right)d\theta$$

The posterior distribution, *p*_*post*(−*i*) (*θ*), is calculated as data minus data point *i*. In the “LOO” package in *R*, *k*-Pareto values are used to compute leave-one-out cross-validation. If the *k* values are below 0.5, the model is generally considered to fit well with the data. However, if the *k* values exceed 0.7, there is a risk of inaccurate estimation.

In MCMC-aided Bayesian analysis, it is important to check if the Markov chain central limit theorem is held, meaning that the iterative samples in a Markov chain are independent. To visually assess the convergence of Markov chains, autocorrelation plots, Gelman–Rubin–Brooks plots, and trace plots are used. The Gelman–Rubin shrink factor (*Rhat*), and the effective sample size (*n_eff*) are used as indicators for the convergence of the chains. The *n_eff* value indicates the number of iterative samples that are not autocorrelated during the stochastic simulation process. If this number is larger than 1000, it is normally considered enough for accurate inference [74]. The *Rhat* value shows the convergence of iterative simulations [75]. If *n_eff* values exceed 1000 and *Rhat* values equal 1, the model is considered well-convergent. The formula for calculating the *Rhat* values [76] is as follows:

$$\hat{R}=\sqrt{\frac{\hat{V}}{W}}$$

where $\hat{R}$ is the *Rhat* value, $\hat{V}$ is the estimated posterior variance, and W is the within-sequence variance.

All analysis steps are conducted on R with the *bayesvl* package [77], which has several advantages, such as open access, good visualization capabilities, and transparent operations. To reduce subjective influences in the estimation due to the exploratory nature of the study, uninformative priors are used. The MCMC setup includes 5000 iterations with 2000 warm-up iterations and 4 chains. Considering the importance of data transparency and the cost of data usage [78,79], all data and code snippets of this study were deposited onto an Open Science Framework server (https://osf.io/bgurv/ (accessed on 30 May 2023)).

## 3. Results

The latest model fitting runs for all models were on 17 May 2023, R version 4.2.1, Windows 11, with a total elapsed time of 63.9 s for Model 1, 45.3 s for Model 2, and 68.8 s for Model 3.

### 3.1. Model 1

**Figure 2:**
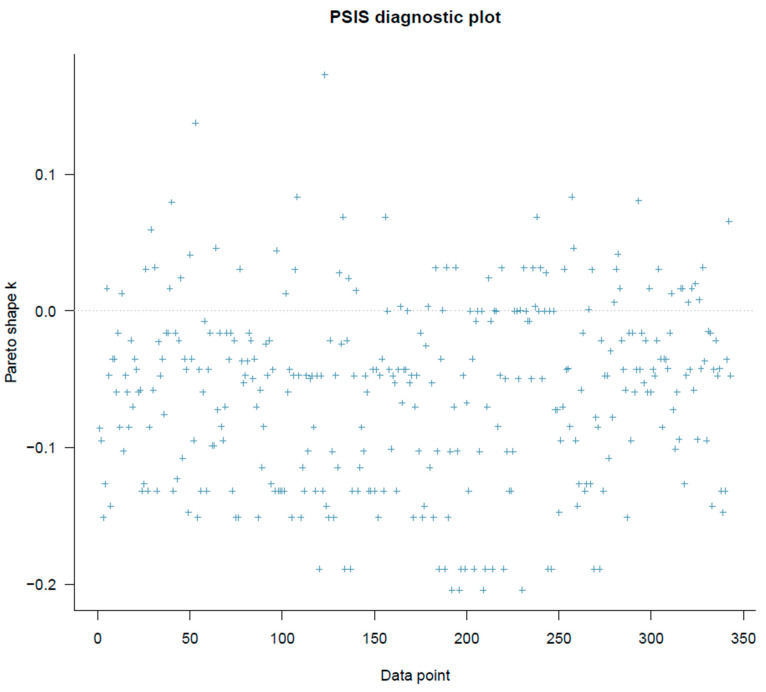
PSIS-LOO diagnostic plot for Model 1.

Figure 2 shows the result of PSIS diagnostics for Model 1. All *k*-values are below the 0.5 threshold, indicating that the model has an acceptable goodness-of-fit.

**Figure 3:**
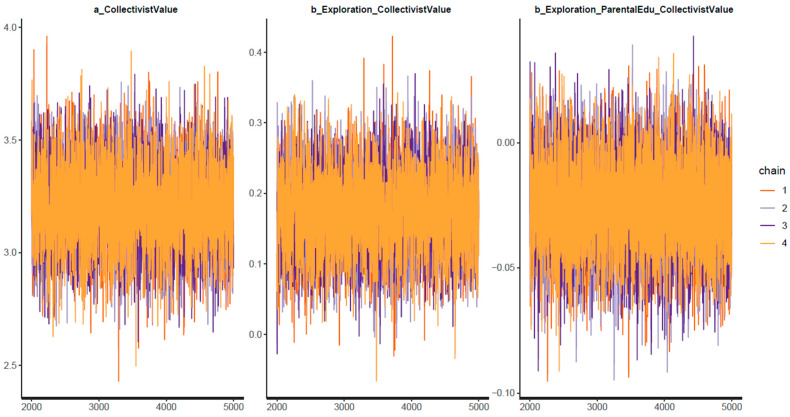
Model 1’s trace plots.

**Figure 4:**
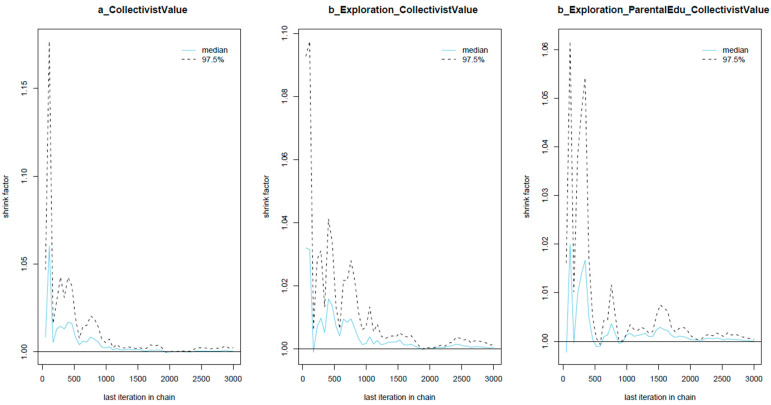
Model 1’s Gelman–Rubin–Brooks plots.

**Figure 5:**
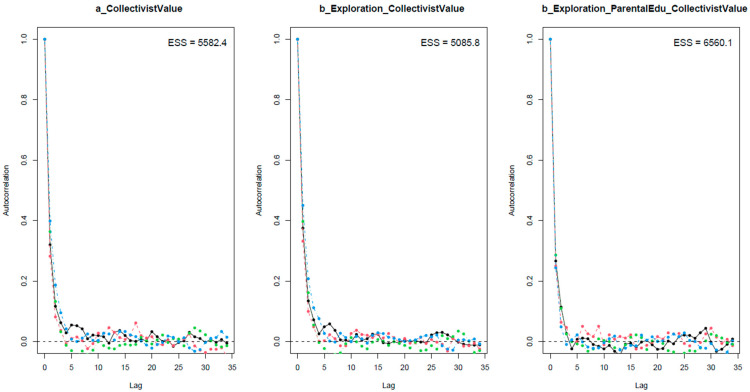
Model 1’s autocorrelation plots.

Table 2 shows the statistical results of the Bayesian analysis for Model 1. For all parameters, the *n_eff* values are above 1000, and the *Rhat* values equal 1, indicating good convergence of the Markov chains. The convergence is also validated visually through the trace plots, the Gelman–Rubin–Brooks plots, and the autocorrelation plots (see Figure 3, Figure 4 and Figure 5, respectively). Figure 3 shows that the chains fluctuate around central equilibriums. Figure 4 shows that the *Rhat* values quickly drop to 1 during the warm-up period. Figure 5 shows that autocorrelation is eliminated early in the estimation process.

**Figure 6:**
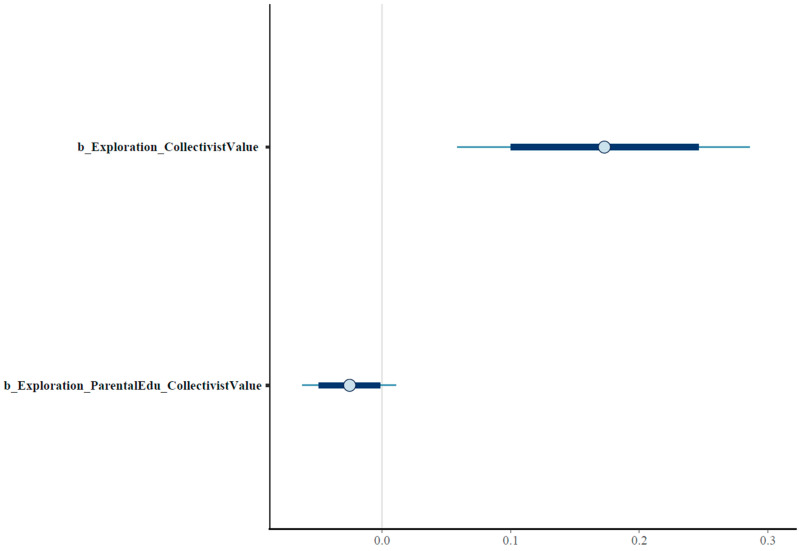
Posterior distributions of Model 1’s coefficients on an interval plot.

Posterior results show that *Exploration* is positively associated with *CollectivisitValue* ($M_{Exploration\;}=0.17$ and $SD_{Exploration\;}=0.06$). *Exploration* ∗ *ParentalEdu* has a slight negative moderating effect ($M_{Exploration*ParentalEdu}=-0.03$ and $SD_{Exploration*ParentalEdu}=0.02$). Note that the magnitude of the moderating effect is small. Figure 6 shows the posterior distrubutions on an interval plot.

**Figure 7:**
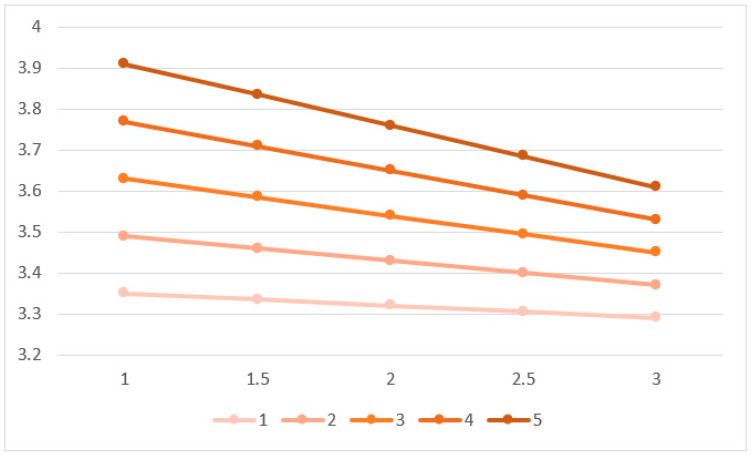
Estimated outcomes based on posterior coefficients.

Figure 7 shows the visualization of estimated degrees of collectivist value integration based on the calculated posterior coefficients. The *y*-axis represents the degree of collectivist value integration. The *x*-axis represents parental education attainment. The line color represents the degree of exploration. Overall, there is a clear difference between the lines regarding their values on the *y*-axis. The lines are downward as parental education attainment is higher, but this difference is relatively small. This downward effect is stronger in higher exploration levels.

### 3.2. Model 2 and Model 3

Similar to the validation processes shown in Model 1, the indicators for Model 2 and Model 3 all show healthy statistical reliability (see Table 3 and Table 4 and Figure A3, Figure A4, Figure A5, Figure A6, Figure A7, Figure A8, Figure A9 and Figure A10 in Appendix A).

**Figure 8:**
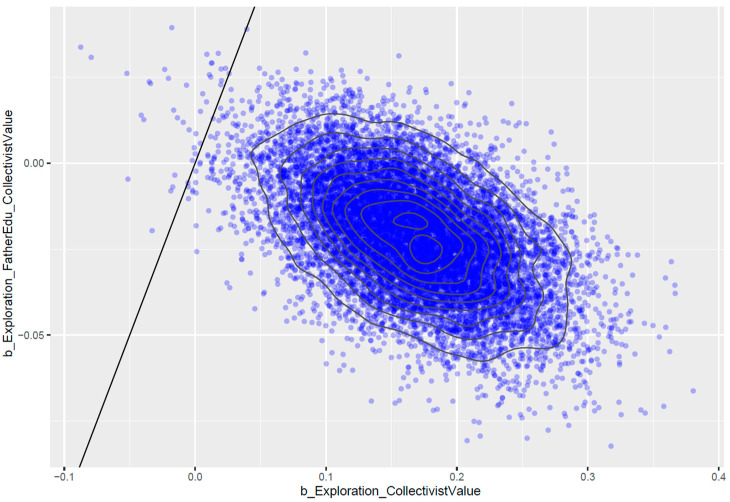
Pairwise posterior distributions of *Exploration* and *Exploration* ∗ *FatherEdu*.

*FatherEdu* has a slight negative moderating effect ($M_{Exploration*FatherEdu}=-0.02$ and $SD_{Exploration*FatherEdu}=0.02$). Figure 8 shows that the posterior distributions of *Exploration* ∗ *FatherEdu* lie mostly on the negative side, indicating that the effect is reliable.

**Figure 9:**
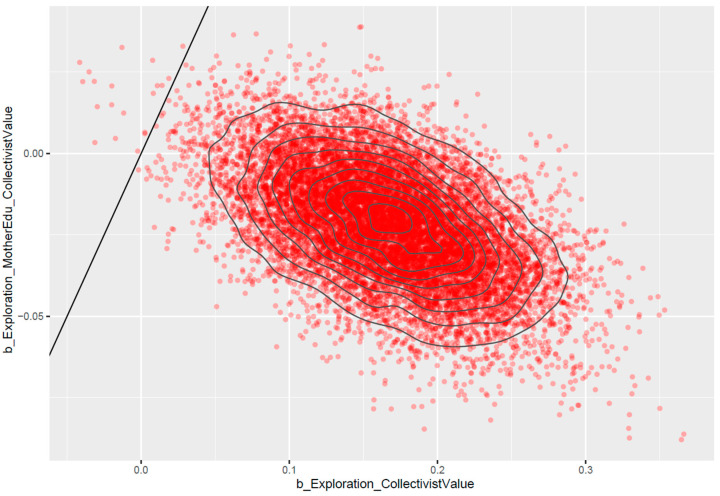
Pairwise posterior distributions of *Exploration* and *Exploration* ∗ *MotherEdu*.

Very similar to the effect of *FatherEdu*, *MotherEdu* also has a slight negative moderating effect ($M_{Exploration*MotherEdu}=-0.02$ and $SD_{Exploration*MotherEdu}=0.02$). Figure 9 shows that the posterior distributions of *Exploration* ∗ *MotherEdu* lie mostly on the negative side, indicating that the effect is reliable.

## 4. Discussion

The finding that students’ exploration would lead to greater prosocial behaviors and more care for collective gains over their own is in alignment with other previous studies [20,21]. From an information-processing viewpoint, the mind performs subjective cost–benefit analyses with the goal of enhancing the system’s perceived advantages and minimizing its perceived expenses. These assessment procedures are based on an individual’s information-processing ability as well as the diversity and openness of their infosphere. Previous studies have demonstrated that an open and diverse infosphere would allow one to access and consequently absorb more information, while better processing capabilities will help one make more accurate assessments of the information [25,58,66]. In this case, exploration is reflected by previous social/natural exploration experiences, which influence how students interpret (make sense of) collectivist values in their minds. Because the mindsponge information processing framework posits that the mind accepts/rejects information based on subjective cost–benefit analysis to sustain the growth and existence of one’s system (represented by the mental construct “self”) [47], more related environmental inputs may shift the perceptions of collectivist values to be more in alignment with self-interests. For example, this kind of processing pathway may contribute to how students interpret common benefits in society through increased sensitivity to group concerns and environmental protection awareness. Altruistic thoughts can be formulated by connecting integrated values in semantic clusters involving many aspects, such as causality, empathy, morality, belongingness, etc., absorbed from prior exploration. Information exchange during exploration processes can happen through social channels, including companionship, family, formal education, the media, digital platforms, etc.

The frequent functioning of these channels (like friendship) can create reinforcing feedback on both the act of exploration and the formed connections of collectivist values, offering a greater level of social support and forming a sense of social connectedness [22,23], which in turn further enhance students’ mental wellness [23,24,58]. Another example is the perceived collective benefits of environmental awareness within the infosphere of society. Related knowledge acquisition, healthy attitude formation, and pro-environmental behavior are all dependent on the interactions between humans and natural elements [25,26]. While being collectivist would also have perceived costs such as conformity to the majority and, therefore, a loss of self-identity, students’ prior living experiences in a historically collectivist infosphere like Chinese society [6] might mitigate the intensity of self-identity-related problems. Thus, this may help increase the likelihood of students incorporating collectivist values in their information interpretation approaches.

The research also suggested that the father and mother’s educational attainment have an equal and slightly negative moderating effect on the association between exploration and collectivist values formation. In other words, as the educational attainment of parents goes higher, the association may become slightly weaker. A possible explanation for such a negative moderating effect is that previous studies have indicated that the higher education attainment the parents receive, the more likely they would be using democratic parenting to educate their children [36,37]. Home scholarly culture is a very important factor in determining students’ perceptions of personal preferences, autonomy, and knowledge sources [55]. Here, the parents are a strong and direct source of input information for a student’s family infosphere. Parenting involving a relatively higher degree of individualistic priority can affect how children interpret information acquired from their external environments. As students learn these pathways of information processing from their parents, strong preconceptions may interfere with the acceptance of collectivist values during the evaluation process. For example, students’ existing strong self-interest prioritization as a result of democratic parenting is likely deemed a conflicting value in relation to newly acquired collectivist values. Based on the mindsponge framework of information processing, such cognitive dissonance could be perceived to be problematic to one’s mental state and decision-making, and consequently would increase the perceived costs of integrating collectivist values. As a result, the association between exploration and collectivist values would be weakened.

**Figure 10:**
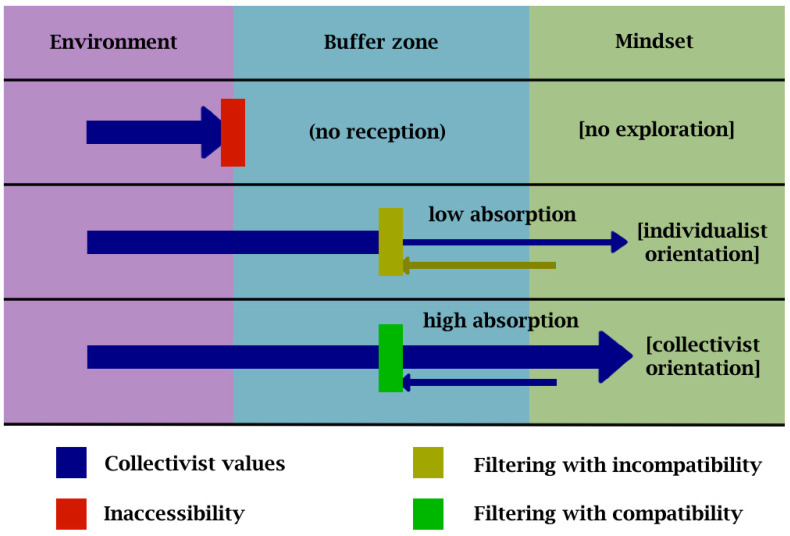
Mindsponge processes of absorbing collectivist values.

Based on the findings of the current study, we can construct conceptual pathways of the filtering processes following the mindsponge framework of information processing. Figure 10 illustrates these pathways in three typical scenarios: no exploration, filtering with incompatible existing values in one’s mindset, and filtering with compatible existing values in one’s mindset. The arrows represent information flows.

If a student does not explore their surrounding environment (upper row in Figure 10), then access to information from these sources is null. Such information includes pieces carrying or being related to collectivist values, and none will be received into the student’s mind. Here, it is worth noting that the attachment of values to a piece of information is heavily subjective for signals requiring higher cognitive processing. If a student has access to information that carries collectivist values but has a rather individualist mindset (middle row in Figure 10), the acceptance rate of such values is low. When the information is filtered within the buffer zone, existing conflicting values from the mindset are used as references and thus lower the net perceived benefit of collectivist values. Individualist values priorly absorbed from interactions with parents decrease the probability of collectivist value acceptance. If a student has access to information that carries collectivist values and has a rather collectivist mindset (lower row in Figure 10), there is relatively less resistance during the filtering process. Here, the collectivist mindset will be easily reinforced with new values in alignment.

## 5. Limitations and Recommendations

The study has several limitations. First of all, the phrase used in the questionnaire 探索周围环境 (exploring the surrounding) include various interactions with different types of environments, and the current study uses the broadest sense of this notion. Further studies may examine more deeply the psychological processes of students’ exploration in each type of specific environment (natural, in-person social, virtual natural, virtual social, etc.). Secondly, there are different cultures and subcultures among Chinese students, so a larger sample size will be more representative. Follow-up studies can examine the same patterns in different study sites, including other areas in China or other countries. Here, the updating manner of Bayesian inference can be advantageous. Thirdly, we did not examine possible specific psychological pathways of students in diverse socioeconomic conditions. Future studies can explore deeper in this direction, and in-depth qualitative approaches may be helpful.

Based on the current study’s findings, there are several recommendations. Firstly, education practitioners and policymakers should encourage extracurricular activities that promote students’ social and physical exploration. These extracurricular activities would enhance information accessibility and acquisition from exploration, providing a better condition for developing a healthy collectivist mindset. Moreover, education practitioners should also forge a supportive environment where individual identity is highly respected. With this support, students who have a stronger sense of self-identity would be less likely to feel threatened in a collectivist environment, which helps lower their perceived costs of integrating collectivist values. Schools should work closely with parents to help students develop a critical and fair analytic mindset that can incorporate collectivist values in alignment with proper perceptions of self-interests.

## 6. Conclusions

Employing MCMC-aided Bayesian analysis on 343 college students in China, we found that, in general, students’ previous exploration of surroundings is positively associated with their acceptance of collectivist values. Parental education attainment moderates this association negatively, but the effect is small in magnitude. This small negative moderating effect is very similar for both the father and mother’s education attainment when examined separately. This study emphasizes two major aspects of information processing in education: information accessibility and information filtering. To absorb more inputs on the interconnection of knowledge in the world, students need to further explore their surroundings. To develop positive perceptions of social values, students need to have proper parental support in value interpretation.

## Figures and Tables

**Table 1:** Variable description.

Variable	Description	Type of Variable	Value
*Exploration*	The students’ level of exploration of the surroundings	Ordinal	Ranging from 1 (not strong) to 5 (very strong)
*ParentalEdu*	The average education attainment level of both parents	Numeric	Ranging from 1 (lowest) to 3 (highest)
*MotherEdu*	Mother’s education attainment	Ordinal	1. Junior middle school or below 2. High school diploma or technical secondary education 3. College degree or above
*FatherEdu*	Father’s education attainment	Ordinal	1. Junior middle school or below 2. High school diploma or technical secondary education 3. College degree or above
*CollectivistValue*	Level of caring about collective gains and losses as much as one’s own	Ordinal	1. Completely disagree 2. Somewhat disagree 3. Difficult to determine 4. Somewhat agree 5. Completely agree

**Table 2:** Model 1’s estimated posteriors.

Parameters	Mean	SD	*n_eff*	*Rhat*
*Constant*	3.21	0.18	5237	1
*Exploration*	0.17	0.06	5249	1
*Exploration* ∗ *ParentalEdu*	−0.03	0.02	7044	1

**Table 3:** Model 2’s estimated posteriors.

	Mean	SD	*n_eff*	*Rhat*
*Constant*	3.21	0.18	6123	1
*Exploration*	0.17	0.06	5545	1
*Exploration* ∗ *FatherEdu*	−0.02	0.02	6843	1

**Table 4:** Model 3′s estimated posteriors.

Parameters	Mean	SD	*n_eff*	*Rhat*
*Constant*	3.20	0.18	5663	1
*Exploration*	0.17	0.06	4772	1
*MotherEdu*	−0.02	0.02	6216	1

**Figure A3:**
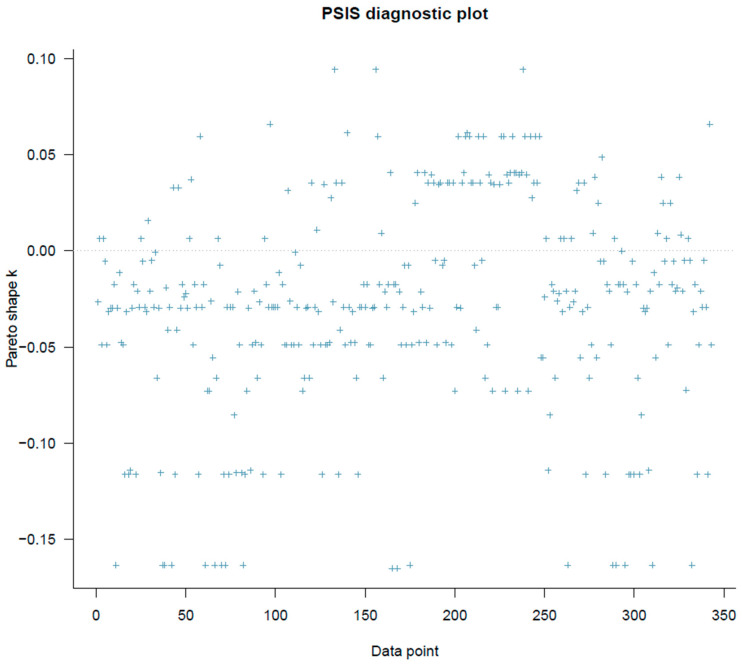
PSIS-LOO diagnostic plot for Model 2.

**Figure A4:**
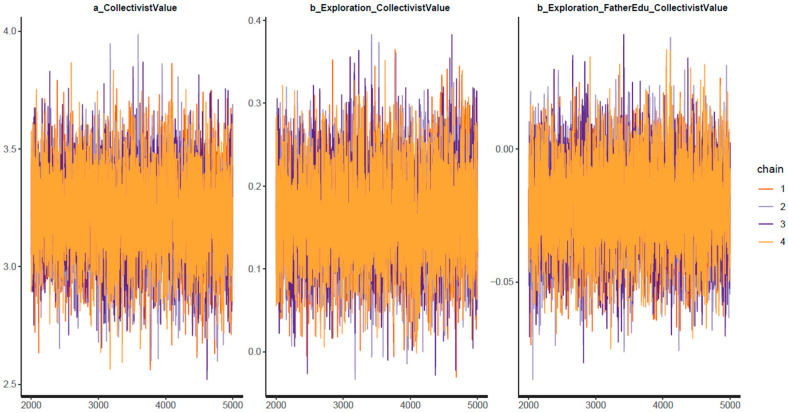
Model 2’s trace plots.

**Figure A5:**
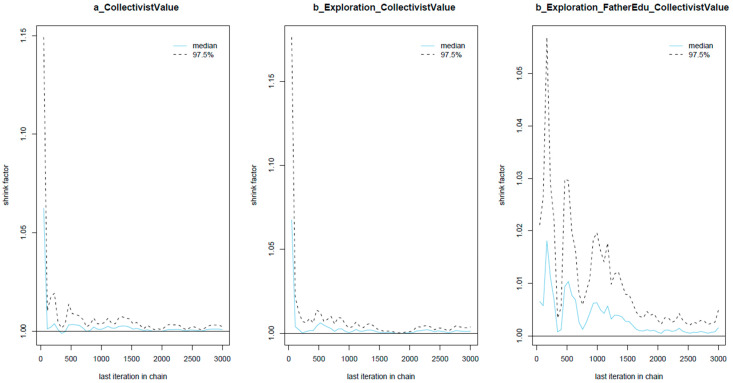
Model 2’s Gelman–Rubin–Brooks plots.

**Figure A6:**
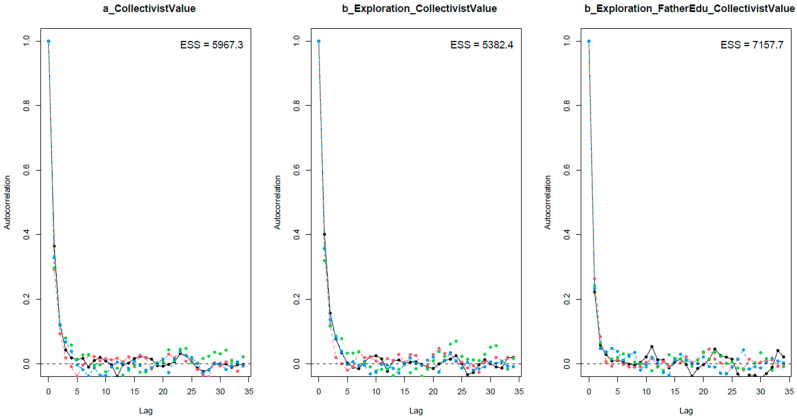
Model 2’s autocorrelation plots.

**Figure A7:**
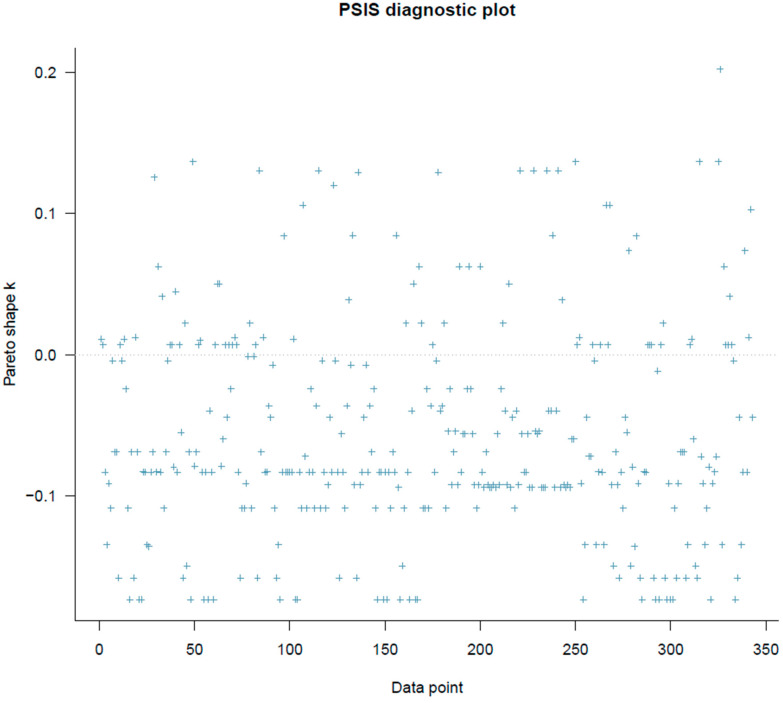
PSIS-LOO diagnostic plot for Model 3.

**Figure A8:**
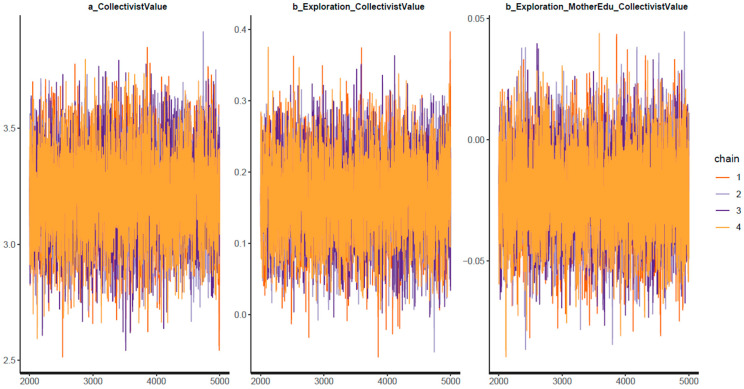
Model 3′s trace plots.

**Figure A9:**
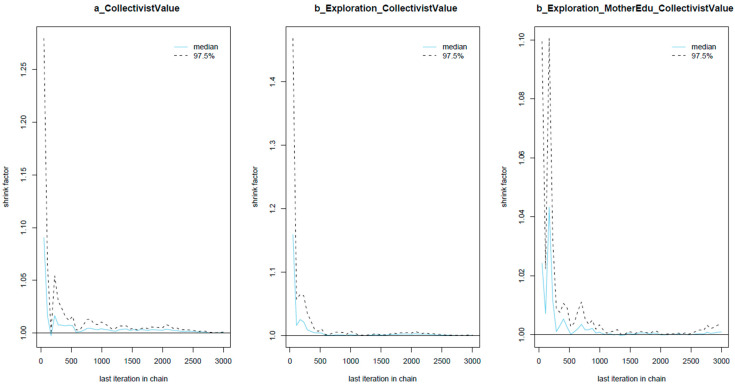
Model 3′s Gelman–Rubin–Brooks plots.

**Figure A10:**
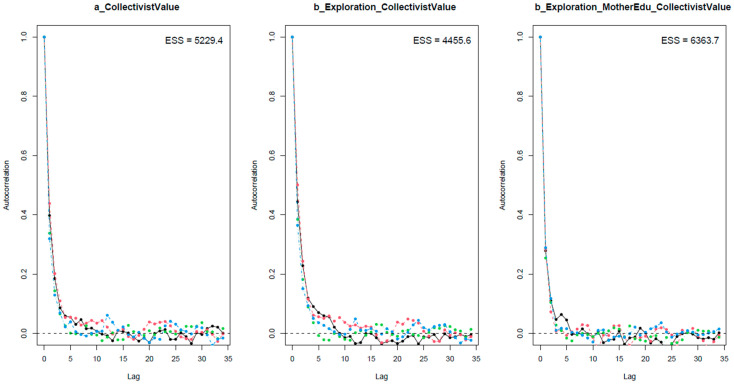
Model 3′s autocorrelation plots.

## Data Availability

All data and code snippets of this study were deposited onto an Open Science Framework server (https://osf.io/bgurv/ (accessed on 30 May 2023)).
